# Supplementary material for: Astrocytes distress triggers brain pathology through induction of δ secretase in a murine model of Alzheimer’s disease
Source: Nat Commun. 2025 Oct 31;16:9653. doi: 10.1038/s41467-025-65536-y (PMC12578805; doi:10.1038/s41467-025-65536-y)

SUPPLEMENTARY INFORMATION

Astrocytes distress triggers brain pathology through induction of δ secretase in a murine model of Alzheimer's disease

Vanessa Schmidt[1]*, Ewelina Ziemlinska[2], Tomasz Obrebski[2], Jemila P Gomes[3], Ewa Zurawska-Plaksej[4], Jaroslaw Cendrowski[5,6], Johan Palmfeldt[7], Barbara L Hempstead[8], Thomas E Willnow[1,3]*, and Anna R Malik[2]*

[1] Max-Delbrueck-Center for Molecular Medicine in the Helmholtz Association, Berlin, Germany

[2] Faculty of Biology, University of Warsaw; Warsaw, Poland

[3] Department of Biomedicine, Aarhus University, Aarhus, Denmark

[4] Department of Toxicology, Wroclaw Medical University, Wroclaw, Poland.

[5] Laboratory of Cell Biology, International Institute of Molecular and Cell Biology, Warsaw, Poland [6] Department of Experimental Oncology, Maria Sklodowska-Curie National Research Institute of Oncology, Warsaw, Poland

[7] Department of Clinical Medicine, Aarhus University, Denmark

[8] Weill Cornell Medical College, New York, USA

SUPPLEMENTARY FIGURES

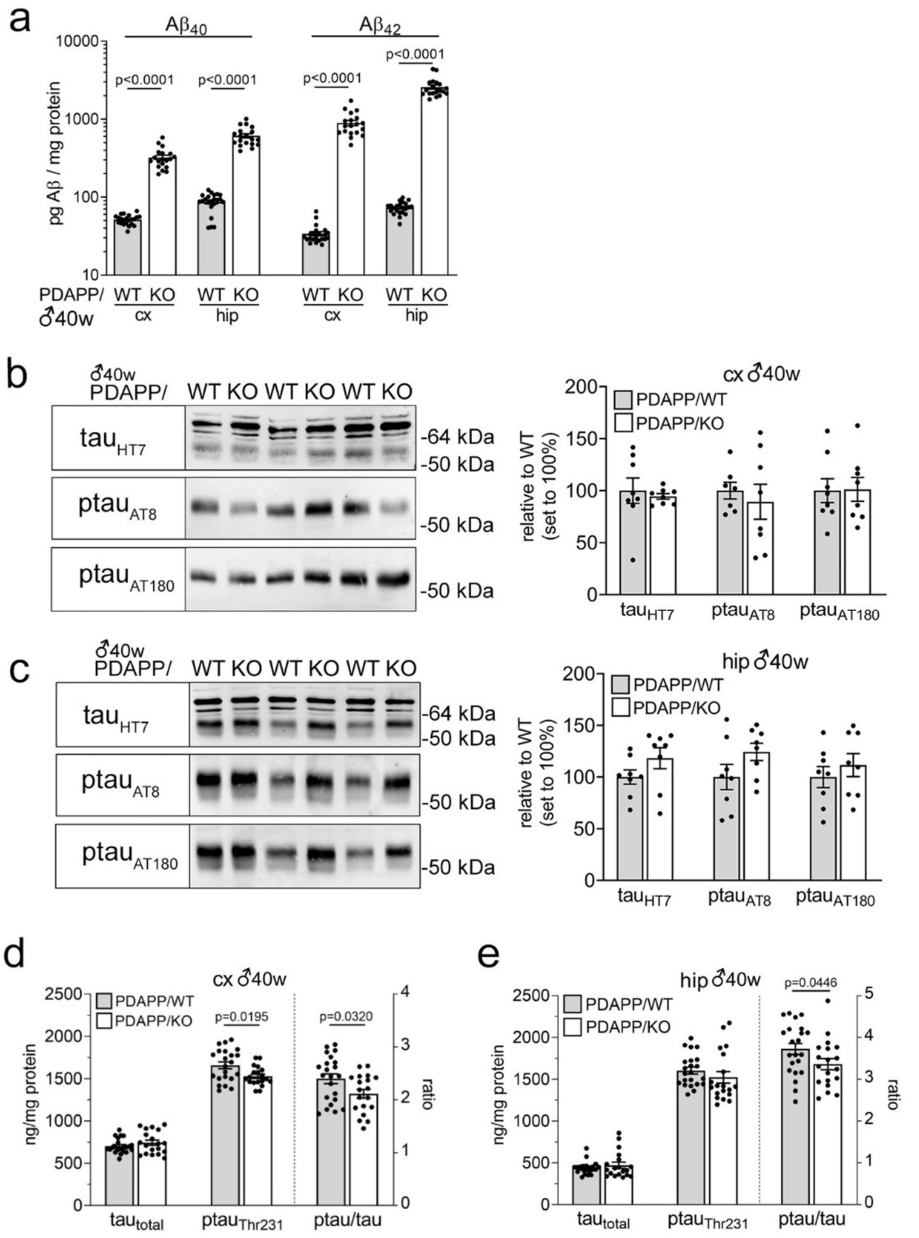

Supplementary Figure 1. Amyloid and tau phenotypes in male PDAPP mice lacking SORCS2

a, Levels of soluble $A\beta_{40}$ and $A\beta_{42}$ in cortex (cx) and hippocampus (hip) of 40 weeks old PDAPP/WT and PDAPP/KO males. Data are given as mean ± SEM. n=22 (WT) and n=19 (KO) animals per group. Data were analysed with unpaired two-sided Mann-Whitney U-test. **b-c**, Representative Western blots (left panels), and densitometric quantification of replicate blots (right panels), documenting levels of total tau (HT7) as well as phosphorylated variants $ptau_{Ser202/Thr205}$ (AT8) and $ptau_{Thr231}$ (AT180) in cx **(b)**

and hip **(c)** of 40 weeks old PDAPP/WT and PDAPP/KO males. Data are expressed as relative to WT (set to 100%) and given as mean ± SEM from n=8 animals per genotype (unpaired two-sided Mann-Whitney U-test or two-sided unpaired Student´s *t*-test). **d-e**, Levels of total (tau_{total}) and phosphorylated variant ($ptau_{Thr231}$) of tau, as well as ratio of $ptau_{Thr231}/tau_{total}$, in cx **(d)** and hip **(e)** of 40 weeks old PDAPP/WT and PDAPP/KO males as measured by ELISA. n=22 (WT) and n=19 (KO) animals per group. Data were analysed with unpaired two-sided Mann-Whitney U-test. p-values for all statistically significant differences are indicated on the graphs. Source data are provided in the Source Data file.

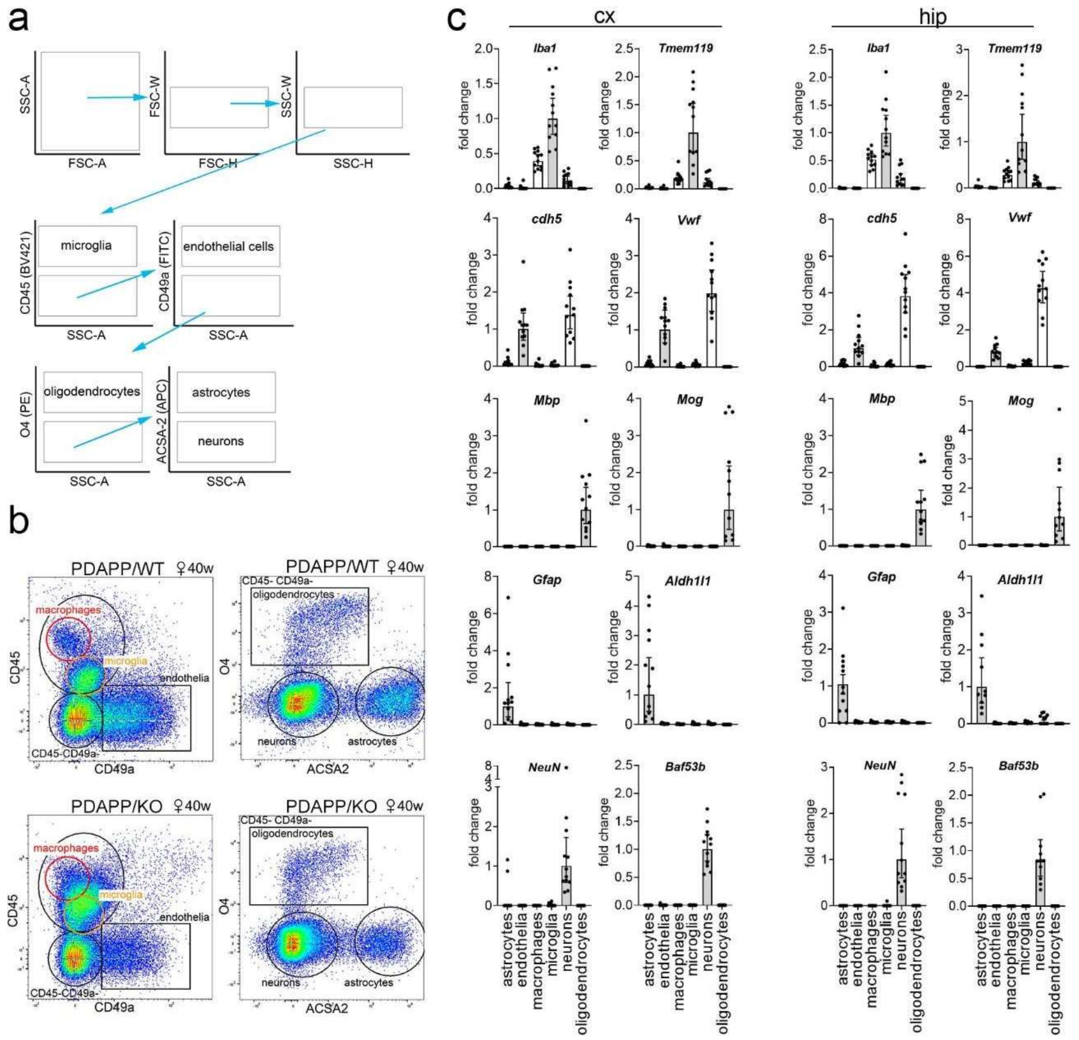

Supplementary Figure 2. Protocol for FACS of cell types from adult mouse brains

a, Gating strategy for multicolor FACS of cells from adult mouse brains. Isolation of cell types was performed using markers CD45+ (microglia, macrophages), CD45- CD49a+ (endothelia), CD45- CD49a- O4+ (oligodendrocytes), CD45- CD49a- O4- ACSA2+ (astrocytes), and CD45- CD49a- O4- ACSA2- (neurons). **b**, Representative FACS panels for cortex from PDAPP/WT and PDAPP/KO females using markers CD45 (macrophages, microglia), CD49a (endothelia), O4 (oligodendrocytes), and ACSA2 (astrocytes). The remaining cell population is enriched in neurons. **c,** The identity of sorted cells from cx and hip samples (pool of PDAPP/WT and PDAPP/KO) was established using qRT-PCR. Markers identify astrocytes (*Gfap, Aldh1l1*), endothelia (*cdh5, Vwf*), macrophages/microglia (*Iba1, Tmem119*), oligodendrocytes (*Mbp, Mog*), and neurons (*NeuN, Baf53b*). *Gapdh* was used as internal control. In each cell population, transcript levels of the markers are given as relative to the marker gene symbolized by the grey bar (*e.g., Iba1* in microglia). Data are given as mean ± SEM for n=12 animals per genotype. Source data are provided in the Source Data file.

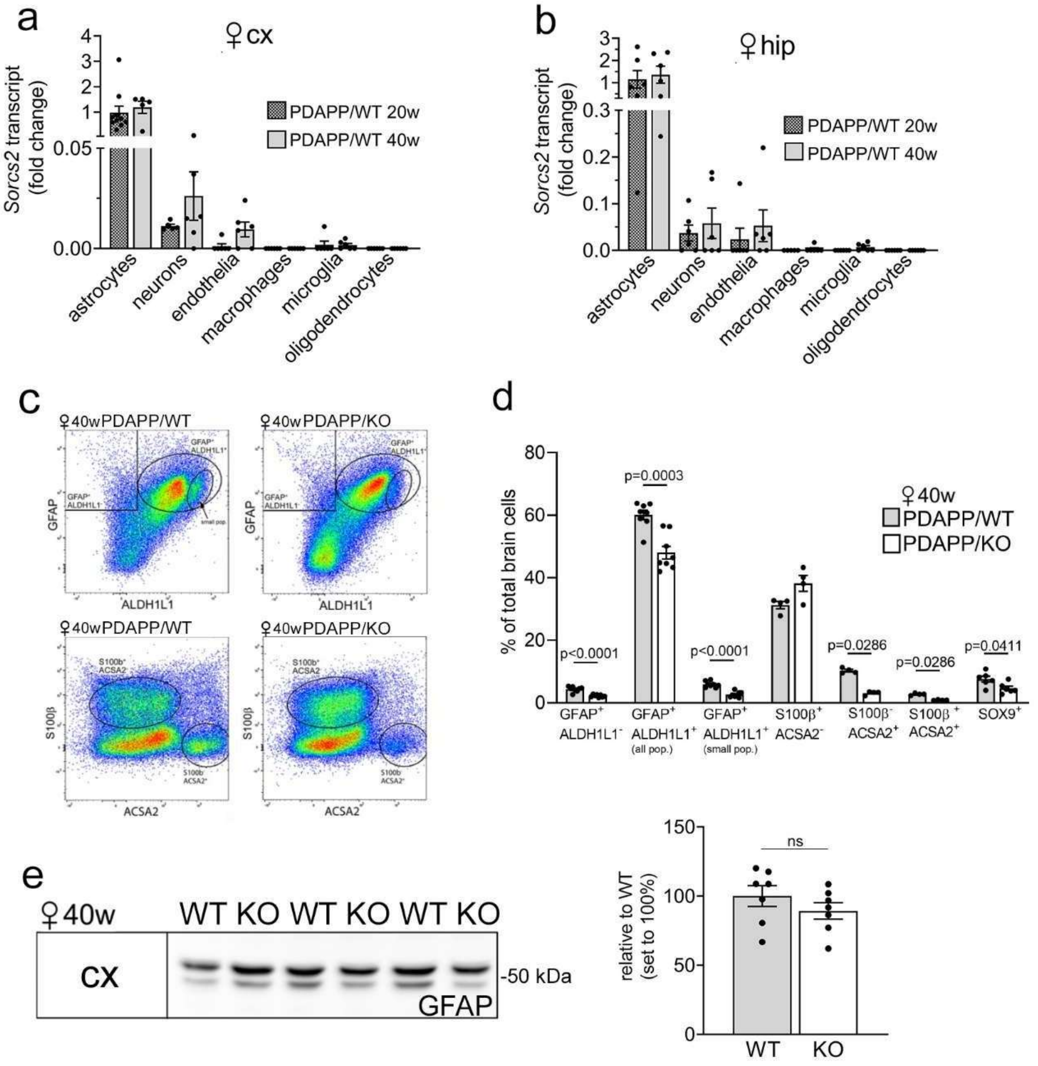

Supplementary Figure 3. Aβ induces loss of astrocytes in SORCS2-deficient mice

a-b, Transcript levels for *Sorcs2* in the indicated cell types isolated by FACS from cx (a) and hip (b) of PDAPP/WT females at 20 and 40 weeks of age. Data are given as mean ± SEM from n=10 (cx, astrocytes 20 w.o.) and n=6 (other groups) animals per group (two-sided unpaired Mann-Whitney U test). **c-d**, Exemplary dot plots (c), and quantifications thereof (d), showing FACS staining profiles and gating (black boxes or circles) of astrocyte subpopulations in cortices of 40 weeks old PDAPP/WT and PDAPP/KO mice using markers GFAP, ALDH1L1, S100b, ACSA2, and SOX9. Data in d are given as mean ± SEM. n=4 animals per genotype in S100b+/ACSA2-, S100b-/ACSA2+, S100b+/ACSA2+; n=6 animals per genotype for SOX9+; n=8 animals per genotype for GFAP+/Aldh1l1-, GFAP+/Aldh1l1+ (all), GFAP+/Aldh1l1+ (small pop.). Data were analysed with two-sided unpaired Student´s *t*-test for fractions stained with GFAP, and two-sided unpaired Mann-Whitney U test for fractions stained with S100b and SOX9. **e**, Representative Western blot for GFAP in cx extracts (left panel), and

quantification from densitometric scanning of replicate blots (right panel), showing comparable levels in 40 weeks old WT and KO mice lacking PDAPP. Data are expressed as relative to WT (set to 100%) and given as mean ± SEM for n=7 animals per genotype (two-sided unpaired Mann-Whitney U test). p-values for all statistically significant differences are indicated on the graphs. ns – not significant. Source data are provided in the Source Data file.

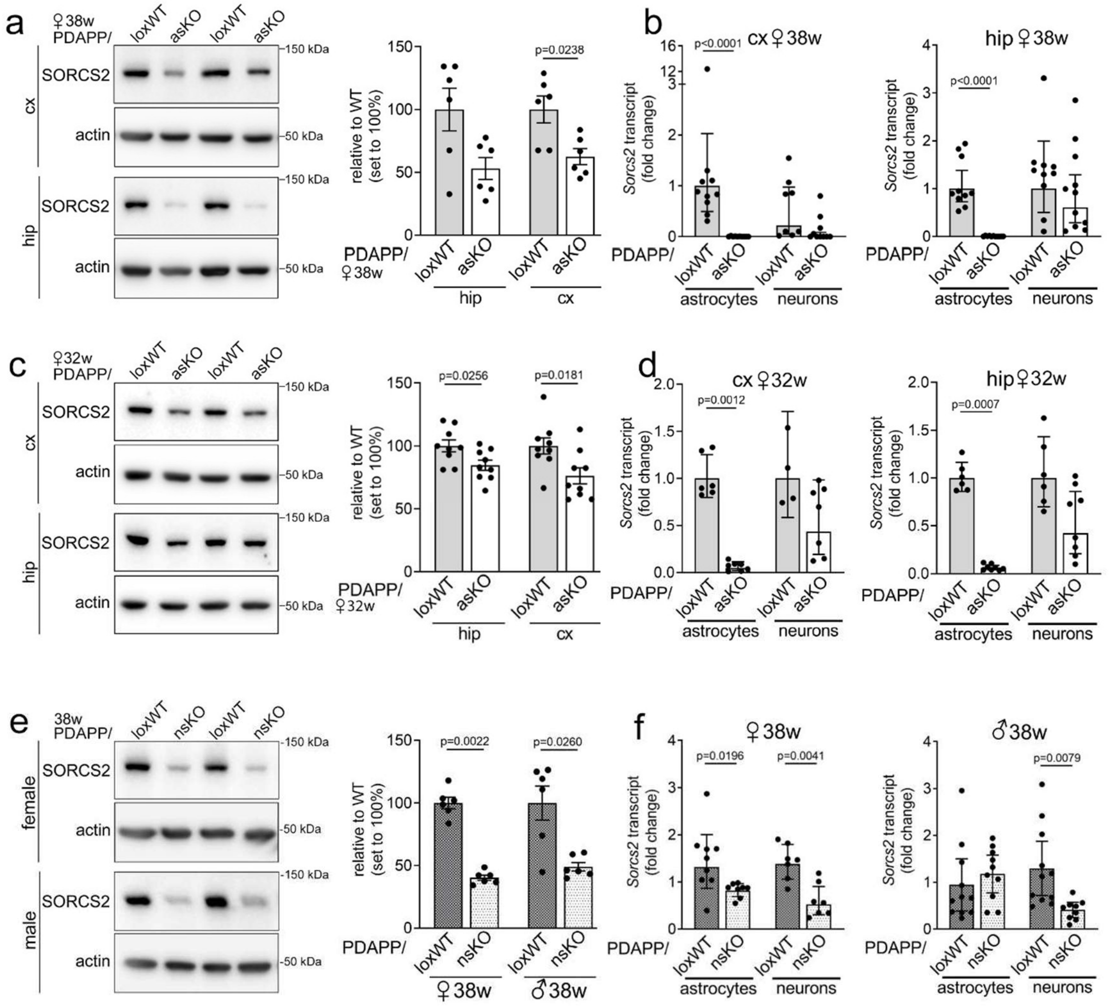

Supplementary Figure 4. Validation of mouse models carrying astrocyte- or neuron-specific *Sorcs2* defects

a, Western blot analyses of cortical (cx) or hippocampal (hip) brain extracts document reduction in SORCS2 levels in female PDAPP mice with astrocyte-specific *Sorcs2* defect (PDAPP/asKO) as compared to PDAPP/loxWT. Animals were 38 weeks of age with time of Cre-ERT2 induction at 26 weeks of age. Exemplary blots as well as quantifications from densitometric scanning of replicate blots are shown. Detection of actin served as loading control. Data are expressed as relative to WT (set to 100%) and are given as mean ± SEM from n=6 animals per genotype (two-sided unpaired Mann-Whitney U-test). **b**, Levels of *Sorcs2* transcript in astrocytes and neurons sorted from cx and hip of PDAPP/loxWT or PDAPP/asKO mice at 38 weeks of age. Loss of *Sorcs2* transcript is seen in astrocytes, but not neurons, in both brain regions. Data are expressed as relative to loxWT (set to 1) and given as mean ± 95% confidence interval. n=8 (cx neurons loxWT); n=10 (cx astrocytes loxWT and asKO; hip astrocytes loxWT; hip neurons loxWT), n=11 (cx neurons asKO; hip astrocytes asKO; hip neurons asKO), Data were anaysed with two-sided unpaired Mann-Whitney U-test). **c-d**, Experiments as in (a-b**)** but testing PDAPP/loxWT and PDAPP/asKO females at 32 weeks of age (induction of Cre-

ERT2 at 20 weeks of age). Data are given as mean ± SEM (c) or mean ± 95% confidence interval (d) from n=4 (cx/neurons/loxWT), n=6 (cx astrocytes loxWT; hip astrocytes loxWT; hip neurons loxWT), n=7 (cx astrocytes asKO), n=8 (hip astrocytes asKO; hip neurons asKO) animals per group (two-sided unpaired Mann-Whitney U-test). **e**, Western blotting and densitometric scanning of replicate blots, show reduction in SORCS2 levels in female and male PDAPP mice with neuron-specific *Sorcs2* defect (PDAPP/nsKO) compared to PDAPP/loxWT animals at 38 weeks of age. Detection of actin served as loading control. Data are expressed as relative to WT (set to 100%), given as mean ± SEM from n=6 animals per genotype (two-sided unpaired Mann-Whitney U-test). **f**, *Sorcs2* transcript levels in astrocytes and neurons sorted from cx of PDAPP/loxWT or PDAPP/nsKO mice at 38 weeks of age. In PDAPP/nsKO males, *Sorcs2* transcript levels are reduced in astrocytes but not neurons, while being reduced in both cell types in PDAPP/nsKO females. Data are expressed as mean ± 95% confidence interval. n=7 (females neurons loxWT and nsKO), n=8 (females astrocytes nsKO), n=9 (females astrocytes loxWT; males neurons nsKO), n=10 (males astrocytes nsKO), n=11 (males astrocytes loxWT, males neurons loxWT) animals per group. Data were analysed by two-sided unpaired Student´s *t*-test. p-values for all statistically significant differences are indicated on the graphs. Source data are provided in the Source Data file.

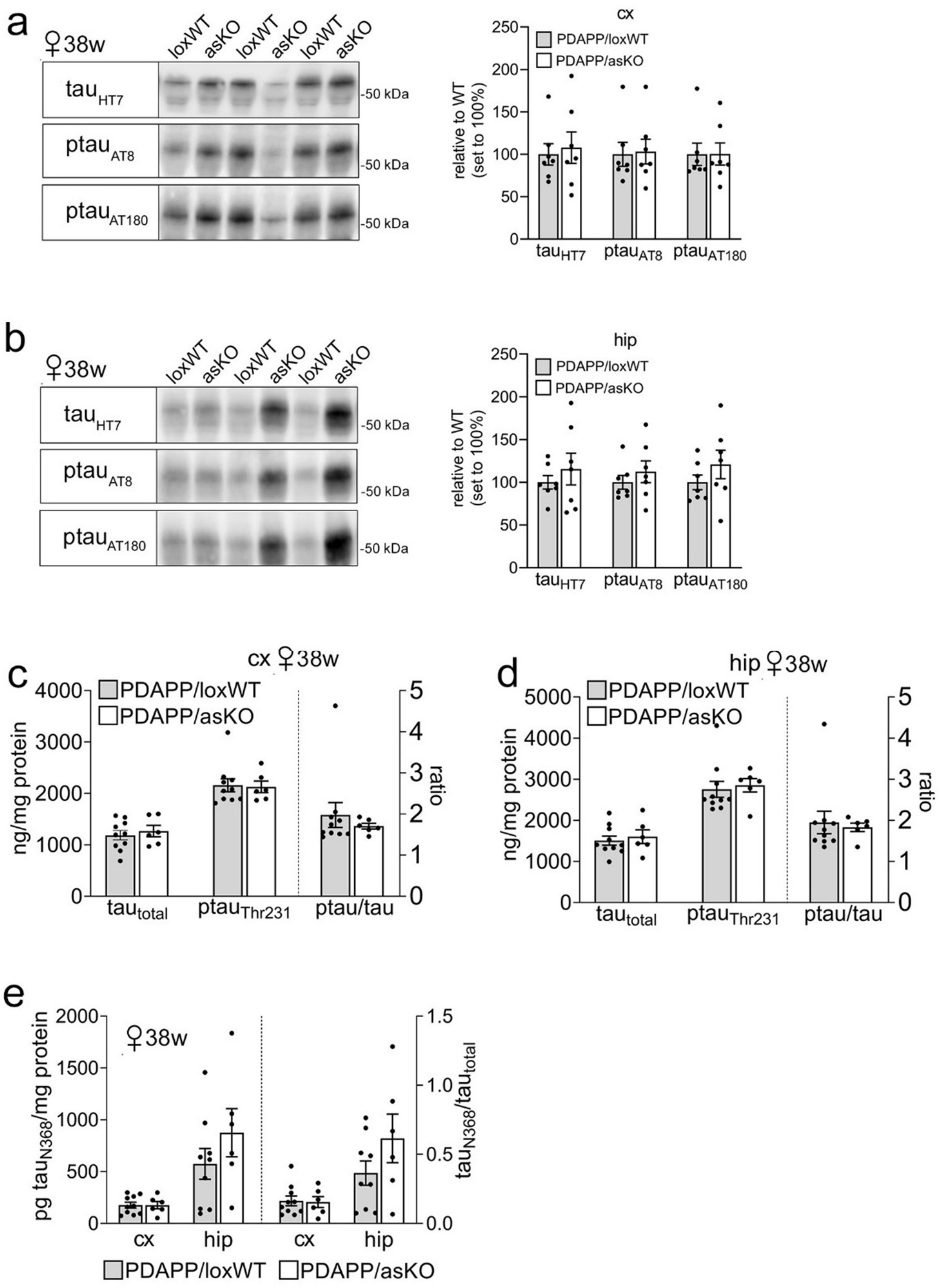

Supplementary Figure 5. Levels of tau in PDAPP mice with astrocyte-specific inactivation of *Sorcs2*

a-b, Representative Western blot analyses (left panels), and densitometric quantification of replicate blots thereof (right panels), document levels of total tau (HT7) as well as phosphorylated variants $ptau_{Ser202/Thr205}$ (AT8) and $ptau_{Thr231}$ (AT180) in cortical (a) and hippocampal (b) extracts of 38 weeks old PDAPP/loxWT or PDAPP/asKO female mice. Data are expressed as relative to WT (set to 100%) and given as mean ± SEM from n=7 animals per genotype (two-sided unpaired Mann-Whitney U-

test). **c-d**, Levels of total (tau_{total}) and $ptau_{Thr231}$, as well as ratio of $ptau_{Thr231}/tau_{total}$, in cortex (c) and hippocampus (d) of 38 weeks old PDAPP/loxWT or PDAPP/asKO female mice determined by ELISA. Data are given as mean ± SEM. n=6 (asKO), n=10 (loxWT) animals per group. Data were analysed with two-sided unpaired Mann-Whitney U-test. **e**, Levels of truncated tau_{N368} and ratio of tau_{N368}/tau_{total}, in cortex and hippocampus of the same animals as in (a-d), determined by ELISA. Data are given as mean ± SEM n=6 (asKO), n=10 (loxWT) animals per group. Data were analysed with two-sided unpaired Mann-Whitney U-test, no statistically significant differences between groups were found. Source data are provided in the Source Data file.

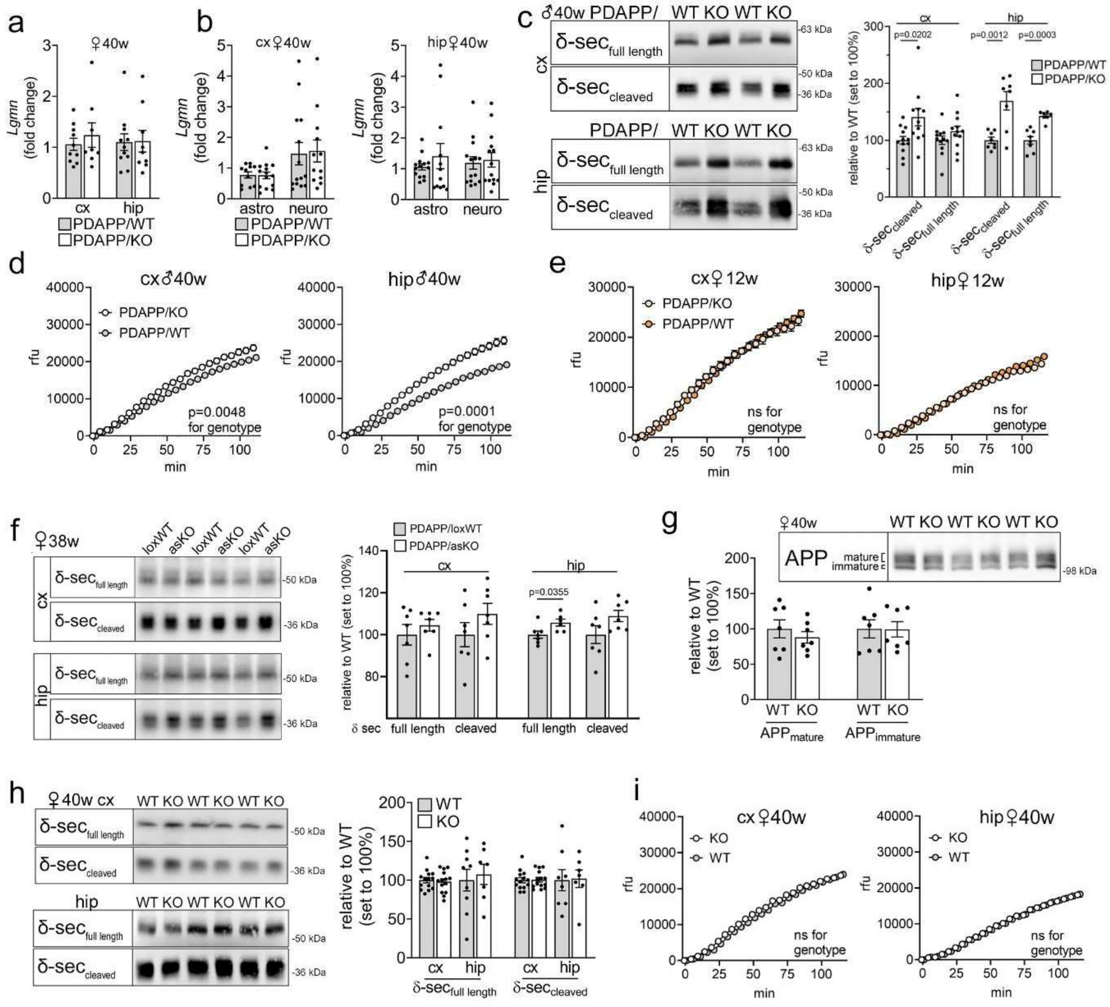

Supplementary Figure 6. Induction of δ secretase expression and activity requires Aβ trigger

a, Transcript levels for d secretase (*Lgmn)* in cx and hip of PDAPP/WT and PDAPP/KO animals. Data are given as mean ± SEM. n=8 (cx KO), n=9 (hip KO), n=10 (cx WT), n=11 (hip WT). Data were analyzed with two-sided unpaired Student´s *t*-test. **b,** Experiment as in **a** but for *Lgmn* transcript levels in the indicated brain cell types isolated by FACS from cx (left panel) and hip (right panel). Data are given as mean ± SEM. n=11 (cx astrocytes WT), n=12 (cx neurons KO; hip astrocytes KO), n=-14 (all other groups). Data were analyzed with two-sided unpaired Mann-Whitney U-test. **c**, Representative Western blot analysis, and densitometric scanning of replicate blots thereof, document increased levels of full-length and cleaved forms of d secretase in cortical (cx) and hippocampal (hip) brain extracts of 40 weeks old PDAPP/KO compared with PDAPP/WT males. Data are expressed as relative to WT (set to 100%) and given as mean ± SEM. n=7 (hip/cleaved/KO), n=8 (hip/cleaved/WT, hip/full/WT and hip/full/KO), n=11 (cx/full/KO); n=12 (cx/cleaved/WT, cx/cleaved/KO and cx/full/WT) animals per group. Data were analyzed with two-sided unpaired Student´s *t*-test. **d**, Activity of d secretase, as

determined by fluorogenic substrate cleavage assay, is significantly increased in cx and hip extracts of 40 weeks old PDAPP/KO as compared to PDAPP/WT male mice. Data are given as mean ± SEM from n=8 (KO) n=9 (WT) animals per group. Data were analyzed with repeated measures two-way ANOVA. **e**, Experiment as in (d) using cx and hip extracts from 12 weeks old PDAPP/WT and PDAPP/KO females. Data show comparable levels of d secretase activity. Data are given as mean ± SEM from n=3 animals per genotype (repeated measures two-way ANOVA). rfu, relative fluorescence units. **f**, Experiment as in (c) quantifying levels of full-length and cleaved forms of d secretase in the cx and hip lysates of 38 weeks old PDAPP/loxWT and PDAPP/asKO females. Data are expressed as relative to WT (set to 100%) and given as mean ± SEM from n=7 animals per genotype (two-sided unpaired Mann- Whitney U test). **g,** Representative Western blot and densitometric scanning of replicate blots, show comparable levels of immature and mature APP in cx extracts of 40 weeks old WT and KO females lacking PDAPP. Data are given as mean ± SEM from n=7 animals per genotype (two-sided unpaired Mann-Whitney U-test). **h**, Experiment as in (c), showing comparable levels of full length and cleaved forms of d secretase in cx and hip extracts of 40 weeks old WT and KO females lacking PDAPP. Data are given as mean ± SEM. n=7 (hip KO), n=8 (hip/cleaved/WT), n=9 (hip/full/WT); n=14 mice (cx) animals per group. Data were analyzed with two-sided unpaired Mann-Whitney U-test for hip, two- sided unpaired Student´s *t*-test for cx. **i,** Experiment as in (d), documenting comparable d secretase activity in cx and hip extracts from 40 weeks old WT and KO females lacking the PDAPP transgene. Data are given as mean ± SEM from n=6 animals per genotype (repeated measures two-way ANOVA). ns, not significant; p-values for all statistically significant differences are indicated on the graphs; rfu, relative fluorescence units. Source data are provided in the Source Data file.

SUPPLEMENTARY TABLES

Supplementary Table 1. Inflammatory profiles of cortical lysates from 20 and 40 weeks old PDAPP/WT and PDAPP/KO females.
Aβ levels determined for all animals are given as concentration range for the respective cohort. Young females were grouped into two cohorts, having modest or high levels of Aβ (cut off: 300 pg/ml $A\beta_{40}$ and 800 pg/ml $A\beta_{42}$ for hip; 35 pg/ml $A\beta_{40}$ and 60 pg/ml $A\beta_{42}$ for cx). Data are given as mean ± SD from n=8-22 animals per genotype (two-sided unpaired Student´s *t*-test; without adjustment for multiple comparisons). ns, not significant; N/A, not available. Markers significantly increased or decreased in PDAPP/KO as compared to matched PDAPP/WT females are highlighted in red or green, respectively. Source data are provided in the Source Data file.

	20 weeks (pg/ml)			40 weeks (pg/ml)		20 weeks (P value)		40 weeks (P value)
	WT	KO (low Aβ)	KO (high Aβ)	WT	KO	WT vs KO (low Aβ)	WT vs KO (high Aβ)	WT vs KO
**pro-inflammatory cytokines / chemokines**								
IL-1b	1.43+0.60	1.84+0.44	0.94+0.41	7.49+4.01	3.60+1.42	0.05	<0.05	<0.0001
TNFa	1.12+0.24	1.58+0.29	1.08+0.20	5.70+4.31	5.78+2.01	<0.001	ns	ns
IL-12p70	69.09+21.74	103.20+26.13	48.71+12.82	103.90+58.87	47.60+15.99	<0.01	<0.01	<0.001
IL-6	48.01+18.84	68.72+14.34	31.52+15.22	15.47+7.80	7.51+2.43	<0.01	<0.05	<0.0001
IP10/CXCL10	3.54+0.47	6.14+2.30	7.80+2.05	21.99+14.62	41.38+12.88	<0.01	<0.0001	<0.0001
MIP1a/CCL3	1.64+0.23	4.33+1.65	5.27+1.16	8.42+6.22	20.76+5.68	<0.001	<0.0001	<0.0001
IL-2	1.94+0.82	2.78+0.56	1.57+0.39	5.39+3.54	2.56+0.84	<0.01	ns	<0.01
IL-5	2.85+0.97	3.97+1.19	1.98+0.88	0.77+0.43	0.37+0.13	<0.05	<0.05	<0.001
IL-16	192.00+29.41	209.70+41.11	277.90+112.0	108.30+82.06	538.3+155.09	ns	<0.05	<0.0001
YKL40/CHI3L1	0.63+0.13	0.93+0.28	1.37+0.36	3.393+2.25	10.47+5.29	<0.0001	<0.0001	<0.0001
**anti-inflammatory cytokines**								
IL-10	2.62+0.85	3.03+0.44	1.95+0.84	7.22+3.40	2.71+1.29	ns	<0.05	<0.0001
IL-4	1.02+0.35	1.22+0.41	0.65+0.28	1.02+0.45	0.38+0.18	ns	<0.01	<0.0001
IL-22	6.87+0.87	6.73+0.65	4.58+0.89	10.48+6.03	5.58+2.18	ns	<0.0001	<0.01
TGFb2	N/A	N/A	N/A	12.24+0.45	9.93+2.11	N/A	N/A	<0.001
TGFb3	N/A	N/A	N/A	1.15+0.21	0.75+0.15	N/A	N/A	<0.0001

UNCROPPED BLOTS FOR SUPPLEMENTARY FIGURES

Uncropped blots for Supplementary Figure 1

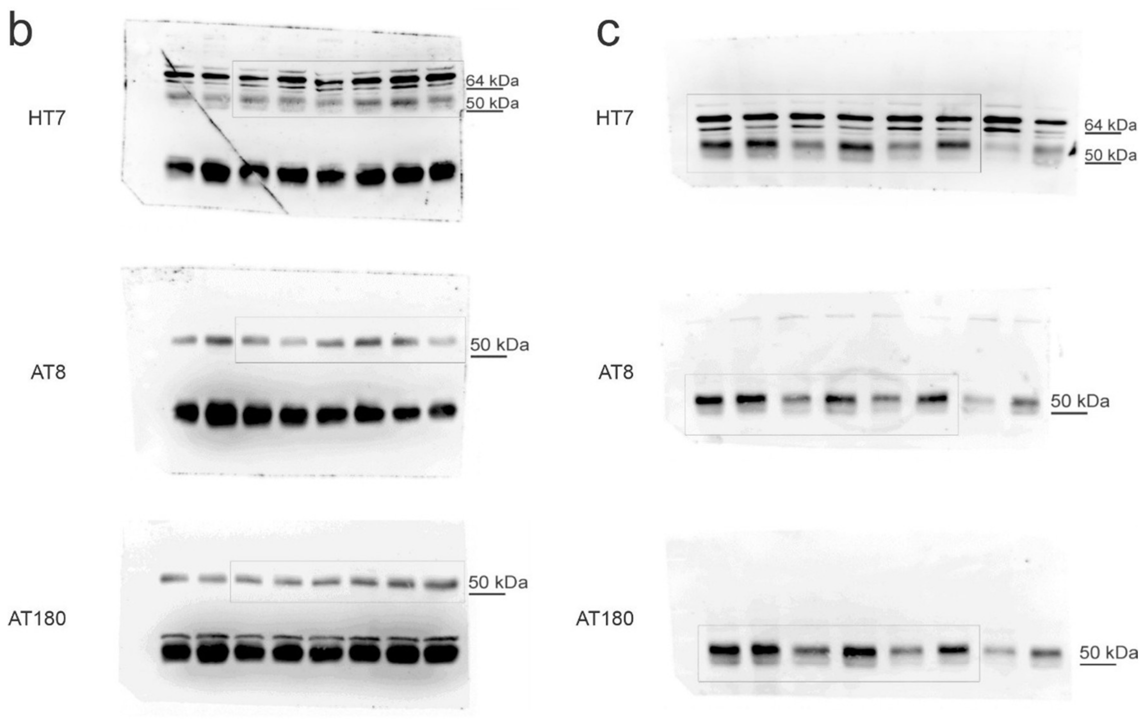

Uncropped blot for Supplementary Figure 3

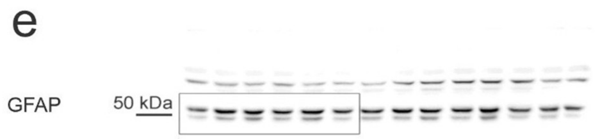

Uncropped blots for Supplementary Figure 4

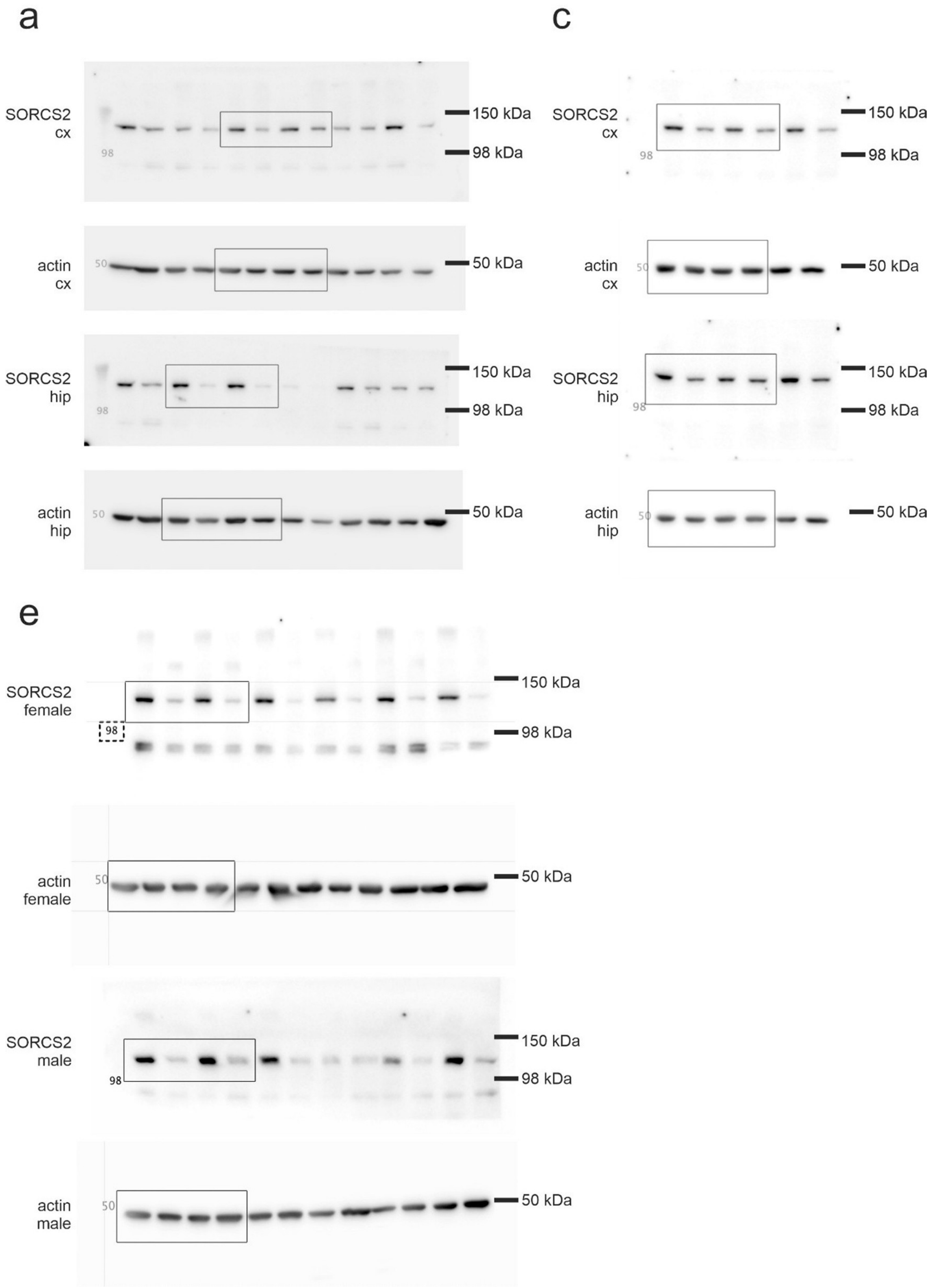

Uncropped blots for Supplementary Figure 5

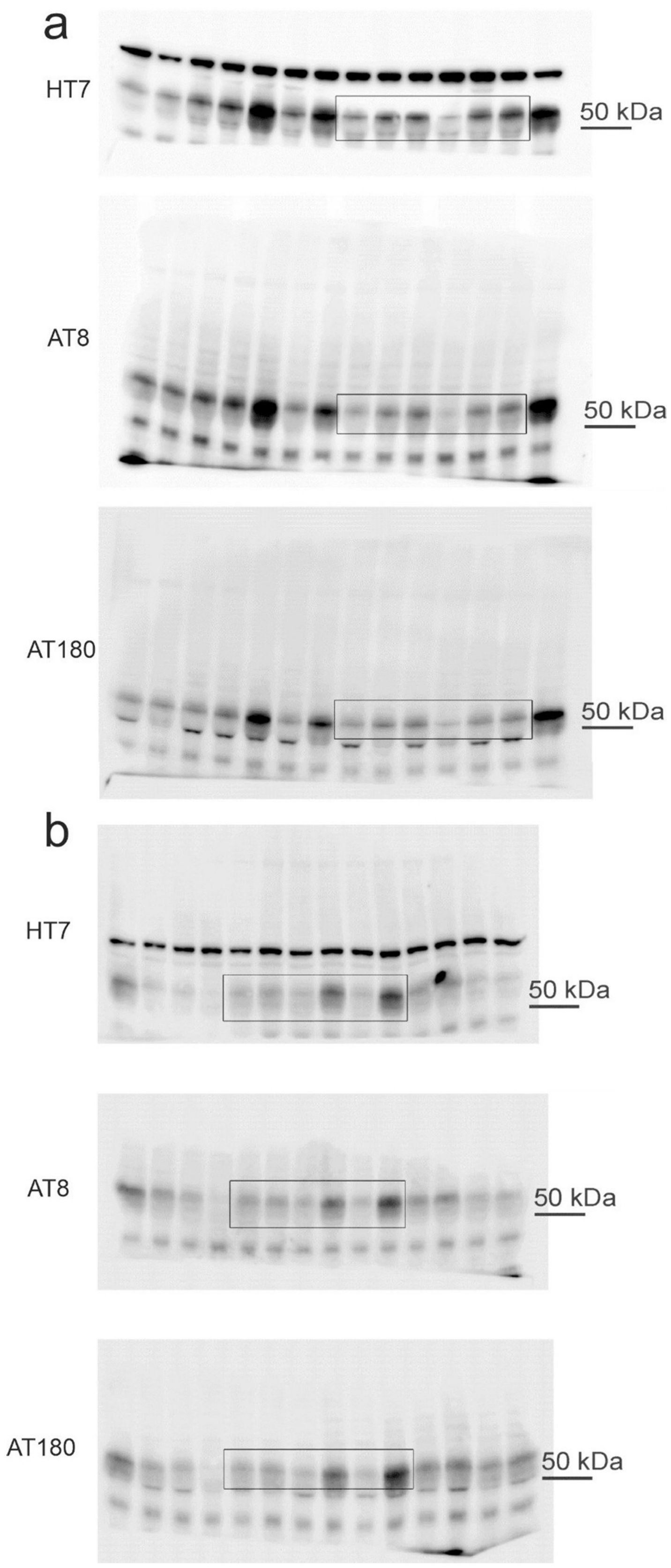

Uncropped blots for Supplementary Figure 6

c

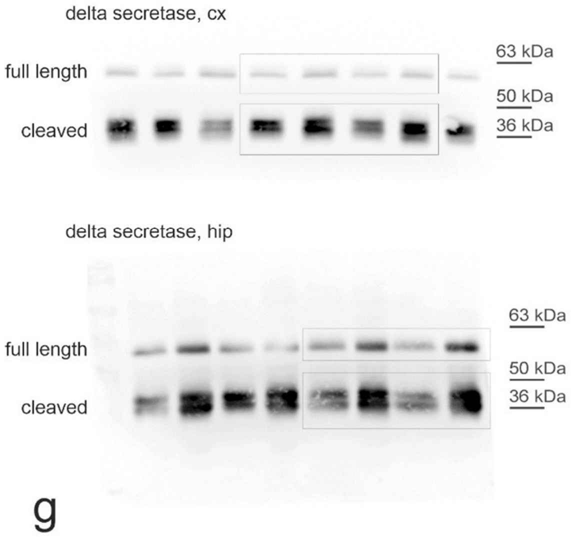

f

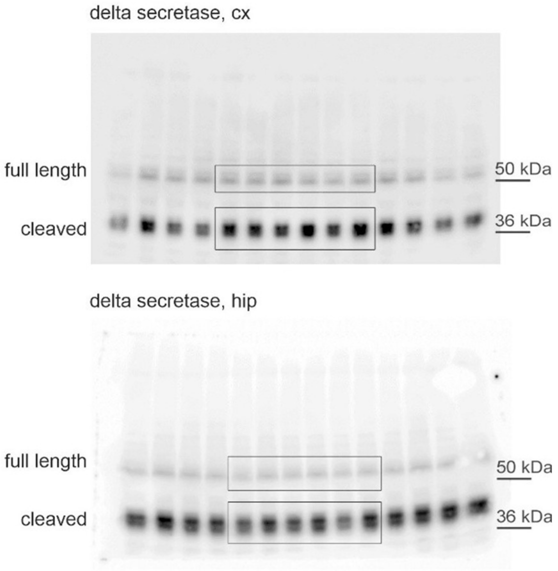

g

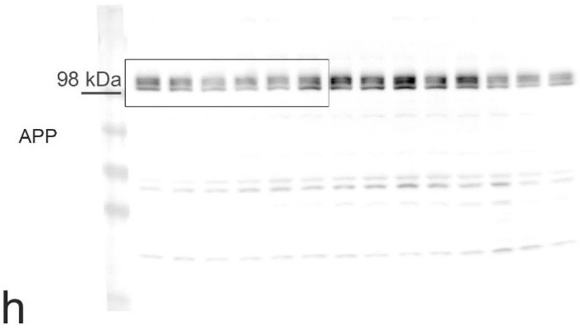

h

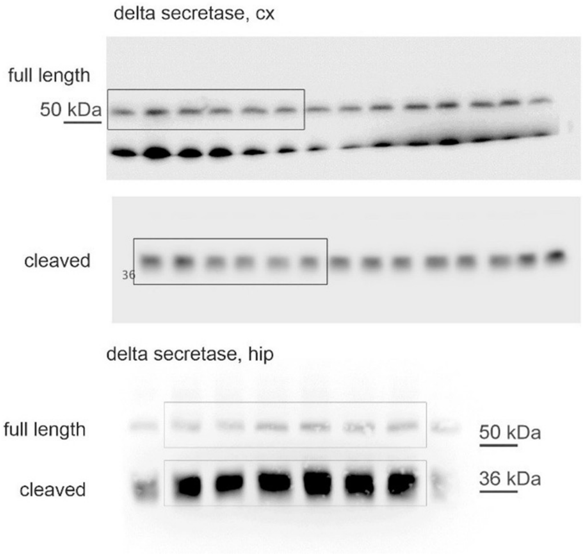

Supplement: Supplementary file 1 — Supplementary Information [file 41467_2025_65536_MOESM1_ESM.pdf]
