# Supplementary material for: Astrocytes distress triggers brain pathology through induction of δ secretase in a murine model of Alzheimer’s disease
Source: Nat Commun. 2025 Oct 31;16:9653. doi: 10.1038/s41467-025-65536-y (PMC12578805; doi:10.1038/s41467-025-65536-y)
Supplement: Supplementary file 2 — Reporting Summary [file 41467_2025_65536_MOESM2_ESM.pdf]

nature portfolio

Corresponding author(s): Vanessa Schmidt, Thomas Willnow, Anna Malik

Last updated by author(s): Sep 24, 2025

Reporting Summary

Nature Portfolio wishes to improve the reproducibility of the work that we publish. This form provides structure for consistency and transparency in reporting. For further information on Nature Portfolio policies, see our Editorial Policies and the Editorial Policy Checklist.

Statistics

For all statistical analyses, confirm that the following items are present in the figure legend, table legend, main text, or Methods section.

n/a	Confirmed	
☐	☒	The exact sample size (n) for each experimental group/condition, given as a discrete number and unit of measurement
☐	☒	A statement on whether measurements were taken from distinct samples or whether the same sample was measured repeatedly
☐	☒	The statistical test(s) used AND whether they are one- or two-sided<br>*Only common tests should be described solely by name; describe more complex techniques in the Methods section.*
☒	☐	A description of all covariates tested
☐	☒	A description of any assumptions or corrections, such as tests of normality and adjustment for multiple comparisons
☐	☒	A full description of the statistical parameters including central tendency (e.g. means) or other basic estimates (e.g. regression coefficient) AND variation (e.g. standard deviation) or associated estimates of uncertainty (e.g. confidence intervals)
☐	☒	For null hypothesis testing, the test statistic (e.g. F, t, r) with confidence intervals, effect sizes, degrees of freedom and P value noted<br>*Give P values as exact values whenever suitable.*
☒	☐	For Bayesian analysis, information on the choice of priors and Markov chain Monte Carlo settings
☒	☐	For hierarchical and complex designs, identification of the appropriate level for tests and full reporting of outcomes
☒	☐	Estimates of effect sizes (e.g. Cohen's d, Pearson's r), indicating how they were calculated

Our web collection on statistics for biologists contains articles on many of the points above.

Software and code

Policy information about availability of computer code

Data collection	Confocal images were acquired using Zeiss LSM 700 and Zen 2012 software. Western blot signals were captured with a Bio-Rad ChemiDoc Imager and Image Lab Touch software (version 2.4.0.03). Lysotracker signal imaging was performed with the Opera Phenix microscope (Revvity). Presto Blue fluorescence measurements were obtained using the VICTOR3 1420 Multilabel Counter (Perkin Elmer) with Victor2030_v4_XP software. Flow cytometry data were collected using Aria Fusion Software (BD). ELISA absorbance measurements were performed using the Mesoscale Discovery platform with Discovery Workbench 4.0.12 and the Tecan Spark 10M plate reader with Spark Control V1.2.20. qPCR data were collected using the StepOnePlus Real-Time PCR System (Applied Biosystems) with StepOne software version 2.2.2.
Data analysis	Western blot analysis was conducted using Image Studio Lite (version 5.2.5) and ImageJ (version 1.52p). Flow cytometry data were analyzed with FlowJo 10. Microscopy images were analyzed with ImageJ (version 1.52p), except for lysotracker signal analysis, which was done using Harmony 4.9 software (Revvity). qPCR data analysis was done with StepOne software version 2.2.2. Mass spectrometry data were analyzed with Thermo Proteome Discoverer 3.0.0.757. Statistical analysis was done in GraphPad Prism 8 or 10.

For manuscripts utilizing custom algorithms or software that are central to the research but not yet described in published literature, software must be made available to editors and reviewers. We strongly encourage code deposition in a community repository (e.g. GitHub). See the Nature Portfolio guidelines for submitting code & software for further information.

Data

Policy information about availability of data

All manuscripts must include a data availability statement. This statement should provide the following information, where applicable:

- Accession codes, unique identifiers, or web links for publicly available datasets
- A description of any restrictions on data availability
- For clinical datasets or third party data, please ensure that the statement adheres to our policy

Source data are provided with this paper. All numerical source data and uncropped images of immunoblots are published alongside the paper as Source Data files. The mass spectrometry data have been deposited to the ProteomeXchange Consortium via the PRIDE partner repository with the dataset identifier PXD067552 (https://www.ebi.ac.uk/pride/archive/projects/PXD067552).

Research involving human participants, their data, or biological material

Policy information about studies with human participants or human data. See also policy information about sex, gender (identity/presentation), and sexual orientation and race, ethnicity and racism.

Reporting on sex and gender	not applicable
Reporting on race, ethnicity, or other socially relevant groupings	not applicable
Population characteristics	not applicable
Recruitment	not applicable
Ethics oversight	not applicable

Note that full information on the approval of the study protocol must also be provided in the manuscript.

Field-specific reporting

Please select the one below that is the best fit for your research. If you are not sure, read the appropriate sections before making your selection.

[x] Life sciences   [ ] Behavioural & social sciences   [ ] Ecological, evolutionary & environmental sciences

For a reference copy of the document with all sections, see nature.com/documents/nr-reporting-summary-flat.pdf

Life sciences study design

All studies must disclose on these points even when the disclosure is negative.

Sample size	No samples size calculations were performed. Sample size and biological replicates for each experiment were described in figure legends and were determined upon consideration of samples availability, duration of experiment and time needed for data processing.
Data exclusions	The data were subjected to ROUT outliers analysis with Q=1% and, where applicable, outliers were removed.
Replication	For in vitro experiments, the findings were confirmed in at least two independent sets of samples. Each set contained samples derived from independent biological replicates, as indicated in figure legends.<br>For in vivo experiments, all data were collected from experiments done on independent biological replicates, each representing an individual mouse, as detailed in the figure legends.
Randomization	No randomization was performed, as there were no experimental setups where individual animals of the same genotype would be subjected to different treatments.
Blinding	For animal studies, the mice were assigned to different groups according to genotypes. The mice were age-and sex-matched between genotypes to minimize variance. All mice were given a number after birth and subsequent experiments were performed blind to genotype as much as possible. For in vitro studies on primary cell preparations, the cultures were assigned to different groups according to the genotypes of the mice and subsequent experiments were performed blind to genotype as much as possible. In case of treatments, cell culture wells assigned to groups randomly.

Reporting for specific materials, systems and methods

We require information from authors about some types of materials, experimental systems and methods used in many studies. Here, indicate whether each material, system or method listed is relevant to your study. If you are not sure if a list item applies to your research, read the appropriate section before selecting a response.

Materials & experimental systems

n/a	Involved in the study
	☒ Antibodies
	☒ Eukaryotic cell lines
☒	☐ Palaeontology and archaeology
	☒ Animals and other organisms
☒	☐ Clinical data
☒	☐ Dual use research of concern
☒	☐ Plants

Methods

n/a	Involved in the study
☒	☐ ChIP-seq
	☒ Flow cytometry
☒	☐ MRI-based neuroimaging

Antibodies

Antibodies used

Anti-MAP2 (D5G1) XP (Cell Signaling Technology, #8707)
Anti-APP antiserum (In-house)
Anti-d-secretase (Legumain) (Cell Signaling Technology, #93627)
Anti-SORCS2 (R&D Systems, #AF4237)
Anti-Iba1 (Abcam, #ab5076; also Wako #019-19741 (IHC))
Anti-GFAP (Sigma, #G3893; also DAKO #Z0334 (#GA524); also Cy3-conjugated Sigma #C9205)
Anti-Cleaved Caspase-3 (Cell Signaling Technology, #9664)
Anti-Cleaved PARP (Asp214) (Cell Signaling Technology, #9541)
Anti-Tau (HT7) (Invitrogen, #MN1000)
Anti-pTau Ser202/Thr205 (AT8) (Invitrogen, #MN1020; biotinylated #MN1020B)
Anti-pTau Thr231 (AT180) (Invitrogen, #MN1040)
Anti-Cleaved tau (tauC3) (Invitrogen, #AHB0061)
Anti-NeuN (biotin-conjugated) (Sigma, #MAB377B)
Anti-S100b (Dako, Agilent, #GA504, ready-to-use)
Anti-S100b-PE (Novus Biologicals, #NBP2-45267)
Anti-SORCS2 (sheep) (R&D Systems, #AF4237)
Anti-CD31 (Novus Biologicals, #NB600-1475)
Anti-Aβ (6E10) (BioLegend, #803014)
Anti-CD45-BV421 (BD Biosciences, #563890)
Anti-CD49a-FITC (Miltenyi Biotec, #130-107-636)
Anti-O4-PE (Miltenyi Biotec, #130-117-357)
Anti-ACSA2-APC (Miltenyi Biotec, #130-116-245)
Anti-GFAP-BV421 (BioLegend, #644710)
Anti-ALDH1L1-FITC (Novus Biologicals, #NBP2-50045F)
Anti-SOX9-488 (Proteintech, #CL488-67439)
Anti-b-actin (Abcam, #ab8227)
Anti-b-actin (Santa-Cruz Biotech, #sc-47778)
Anti–GAPDH (GeneTex, #GTX627408)

Validation

Anti- MAP2 (D5G1) XP (Cell Signaling Technology, #8707): https://www.cellsignal.com/products/primary-antibodies/map2-d5g1-xp-rabbit-mab/8707
Anti-APP antiserum (In-house) DOI: 10.1073/pnas.0503689102
Anti-d-secretase (Legumain) (D6S4H) (Cell Signaling Technology, #93627): https://www.cellsignal.com/products/primary-antibodies/legumain-d6s4h-rabbit-mab/93627
Anti-SORCS2 (R&D Systems, #AF4237): https://www.rndsystems.com/products/mouse-sorcs2-antibody_af4237
Anti-Iba1 (Abcam, #ab5076): https://www.abcam.com/en-us/products/primary-antibodies/iba1-antibody-ab5076
Anti-IBA1 (Wako #019-19741): https://labchem-wako.fujifilm.com/us/product/detail/W01W0101-1974.html
Anti-GFAP (Sigma, #G3893): https://www.sigmaaldrich.com/PL/pl/product/sigma/g3893
Anti-GFAP (DAKO #Z0334 (#GA524)): https://www.agilent.com/en/product/immunohistochemistry/antibodies-controls/primary-antibodies/glial-fibrillary-acidic-protein-(concentrate)-76683
Anti-GFAP Cy3-conjugated (Sigma, #C9205): https://www.sigmaaldrich.com/PL/pl/product/sigma/c9205
Anti-Cleaved caspase-3 (Cell Signaling Technology, #9664): https://www.cellsignal.com/products/primary-antibodies/cleaved-caspase-3-asp175-5a1e-rabbit-mab/9664
Anti-Cleaved PARP (Asp214) (Cell Signaling Technology, #9541): https://www.cellsignal.com/products/primary-antibodies/cleaved-parp-asp214-antibody/9541
Anti-Tau (HT7) (Thermo Fisher/Invitrogen, #MN1000): https://www.thermofisher.com/antibody/product/Tau-Antibody-clone-HT7-Monoclonal/MN1000
Anti-pTau Ser202/Thr205 (AT8) (Thermo Fisher/Invitrogen, #MN1020): https://www.thermofisher.com/antibody/product/Phospho-Tau-Ser202-Thr205-Antibody-clone-AT8-Monoclonal/MN1020
Anti-pTau Ser202/Thr205 (AT8) biotinylated (Thermo Fisher/Invitrogen, #MN1020B): https://www.thermofisher.com/antibody/product/Phospho-Tau-Ser202-Thr205-Antibody-clone-AT8-Monoclonal/MN1020B
Anti-pTau Thr231 (AT180) (Thermo Fisher/Invitrogen, #MN1040): https://www.thermofisher.com/antibody/product/Phospho-Tau-Thr231-Antibody-clone-AT180-Monoclonal/MN1040

Anti-Cleaved tau (tauC3) (Invitrogen, #AHB0061): https://www.thermofisher.com/antibody/product/Tau-Cleaved-Asp421-Asp422-Antibody-clone-TauC3-Monoclonal/AHB0061
Anti-NeuN (biotin-conjugated) (Sigma, #MAB377B): https://www.sigmaaldrich.com/PL/pl/product/mm/mab377b
Anti-S100b (Dako, Agilent, #GA504, ready-to-use): https://www.agilent.com/en/product/immunohistochemistry/antibodies-controls/primary-antibodies/s100-(dako-omnis)-76198
Anti-S100b-PE (Novus Biologicals, #NBP2-45267): https://www.novusbio.com/products/s100b-antibody-15f9nb_nbp2-45267
Anti-SORCS2 (R&D Systems, #AF4237): https://www.rndsystems.com/products/mouse-sorcs2-antibody_af4237
Anti-CD31 (Novus Biologicals, #NB600-1475): https://www.novusbio.com/products/cd31-pecam-1-antibody-mec133_nb600-1475
Anti-Aβ (6E10) (BioLegend, #803014): https://www.biolegend.com/en-ie/products/anti-beta-amyloid-1-16-antibody-10998
Anti-CD45-BV421 (BD Biosciences, #563890): https://www.bdbiosciences.com/en-dk/products/reagents/flow-cytometry-reagents/research-reagents/single-color-antibodies-ruo/bv421-rat-anti-mouse-cd45.563890?tab=product_details
Anti-CD49a-FITC (Miltenyi Biotec, #130-107-636): https://www.miltenyibiotec.com/DE-en/products/cd49a-antibody-anti-mouse-rat-reafinity-rea493.html
Anti-O4-PE (Miltenyi Biotec, #130-117-357): https://www.miltenyibiotec.com/US-en/products/o4-antibody-anti-human-mouse-rat-o4.html
Anti-ACSA2-APC (Miltenyi Biotec, #130-116-245): https://www.miltenyibiotec.com/US-en/products/acsa-2-antibody-anti-mouse-reafinity-rea969.html
Anti-GFAP-BV421 (BioLegend, #644710): https://www.biolegend.com/nl-nl/products/brilliant-violet-421-anti-gfap-antibody-12793
Anti-ALDH1L1-FITC (Novus Biological, #NBP2-50045F): https://www.novusbio.com/products/aldh1l1-antibody-4a12_nbp2-50045
Anti-SOX9-488 (Proteintech, #CL488-67439): https://www.ptglab.com/products/SOX9-Antibody-CL488-67439.htm
Anti-b-actin (Abcam, #ab8227): https://www.abcam.com/en-us/products/primary-antibodies/beta-actin-antibody-loading-control-ab8227
Anti-b-actin (Santa-Cruz Biotech, #sc-47778): https://www.scbt.com/p/beta-actin-antibody-c4
Anti–GAPDH (GeneTex, #GTX627408) https://www.genetex.com/Product/Detail/GAPDH-antibody-GT239/GTX627408

Eukaryotic cell lines

Policy information about cell lines and Sex and Gender in Research

Cell line source(s)	SY5Y cells used in this study were obtained from ATCC (https://www.atcc.org/)
Authentication	not applicable; cell line was bought from ATCC
Mycoplasma contamination	Cell lines were routinely tested for mycoplasma contamination and were negative.
Commonly misidentified lines (See ICLAC register)	The cell line used in this study (SH-SY5Y) is not on the list of misidentified cell lines.

Animals and other research organisms

Policy information about studies involving animals; ARRIVE guidelines recommended for reporting animal research, and Sex and Gender in Research

Laboratory animals	Mice
Wild animals	not applicable
Reporting on sex	Both sexes were used in this study as detailed in the manuscript.
Field-collected samples	not applicable
Ethics oversight	All animal experimentations were performed according to institutional guidelines following approval by authorities of the State of Berlin (X9007/17; X9009/22; G0105/22) or the First Ethical Committee Warsaw (1375P1/2022).

Note that full information on the approval of the study protocol must also be provided in the manuscript.

Plants

Seed stocks	not applicable
Novel plant genotypes	not applicable
Authentication	not applicable

Flow Cytometry

Plots

Confirm that:

☐ The axis labels state the marker and fluorochrome used (e.g. CD4-FITC).

☒ The axis scales are clearly visible. Include numbers along axes only for bottom left plot of group (a 'group' is an analysis of identical markers).

☐ All plots are contour plots with outliers or pseudocolor plots.

☐ A numerical value for number of cells or percentage (with statistics) is provided.

Methodology

Sample preparation	Sample preparation is detailed in the Methods section of the manuscript
Instrument	BD Aria Fusion
Software	BD FACS Diva Software and FlowJo V10
Cell population abundance	the purity of the sorted fractions was confirmed by qPCR (2 different markers for each cell population)
Gating strategy	There are example images in the supplementary material with delineation of the gates

☒ Tick this box to confirm that a figure exemplifying the gating strategy is provided in the Supplementary Information.
